# Phylostems: a new graphical tool to investigate temporal signal of heterochronous sequences datasets

**DOI:** 10.1093/bioadv/vbad026

**Published:** 2023-03-13

**Authors:** Anna Doizy, Amaury Prin, Guillaume Cornu, Frederic Chiroleu, Adrien Rieux

**Affiliations:** CIRAD, UMR PVBMT, La Réunion, St Pierre 97410, France; DoAna—Statistiques Réunion, Reunion Island, Saint-Joseph F-97480, France; CIRAD, UMR PVBMT, La Réunion, St Pierre 97410, France; CIRAD, Univ Montpellier, UR Forests and Societies, 34398 Montpellier Cedex 5, France; CIRAD, UMR PVBMT, La Réunion, St Pierre 97410, France; CIRAD, UMR PVBMT, La Réunion, St Pierre 97410, France

## Abstract

**Motivation:**

Molecular tip-dating of phylogenetic trees is a growing discipline that uses DNA sequences sampled at different points in time to co-estimate the timing of evolutionary events with rates of molecular evolution. Importantly, such inferences should only be performed on datasets displaying sufficient temporal signal, a feature important to test prior to any tip-dating inference. For this purpose, the most popular method considered to-date has been the ‘root-to-tip regression’ which consist in fitting a linear regression of the number of substitutions accumulated from the root to the tips of a phylogenetic tree as a function of sampling times. The main limitation of the regression method, in its current implementation, relies in the fact that the temporal signal can only be tested at the whole-tree scale (i.e. its root).

**Results:**

To overcome this limitation we introduce Phylostems, a new graphical user-friendly tool developed to investigate temporal signal within every clade of a phylogenetic tree. We provide a ‘how to’ guide by running Phylostems on an empirical dataset and supply guidance for results interpretation.

**Availability and implementation:**

Phylostems is freely available at https://pvbmt-apps.cirad.fr/apps/phylostems.

## 1 Introduction

‘Tip-dating’ of phylogenetic trees is a popular and powerful type of inference aiming to make use of sequence data isolated at different points in time (i.e. heterochronous datasets) to co-estimate the timing of evolutionary events with rates of molecular evolution (Rieux and Balloux, 2016). As a prerequisite, tip-dating requires working on measurably evolving populations (MEPs) which consist in datasets displaying detectable amounts of *de novo* nucleotide changes among the DNA sequences sampled at different timepoints (Drummond *et al.*, 2003b). Such phylogenetic inferences represent a powerful tool for biological hypothesis testing and have notably been critical for *(i)* dating key events in human evolutionary history, *(ii)* improving our understanding of various important pathogens emergence, spread and evolution, *(iii)* investigating the relative impacts of climatic and anthropogenic factors on the widespread extinctions of large mammals, *(iv)* providing meaningful information about pathogens host species jumps and *(v)* estimating unknown sequence’s ages in various organisms [see Rieux and Balloux (2016) and references herein for review].

Tip-dating inferences should only be performed when there is sufficient temporal signal within the analysed dataset (Drummond *et al.*, 2003b; Duchêne *et al.*, 2015; Murray *et al.*, 2016; Rieux and Balloux, 2016), a feature which might not be the case if *(i)* the sampling period is too short, *(ii)* evolutionary rates are too low or variable amongst lineages or *(iii)* some samples have incorrectly been dated (Rambaut *et al.*, 2016). As such it is important for researchers to be able to test their dataset for the amount and consistency of temporal signal prior to any tip-dating inference. For this purpose, the most popular method considered to-date has been the ‘root-to-tip regression’ which consist in fitting a linear regression of the number of substitutions accumulated from the root to the tips of a phylogenetic tree as a function of sampling times (Buonagurio *et al.*, 1986; Drummond *et al.*, 2003a; Korber *et al.*, 2000; Shankarappa *et al.*, 1999). If sampling dates are sufficiently different, then more recently sampled sequences should have undergone substantially more evolutionary change than earlier sampled sequences, which would result in a positive correlation slope. This method has often been used as a diagnostic of data quality and of the reliability rate estimates, where the slope coefficient corresponds to the substitution rate under the assumption of a strict molecular clock, the *X*-intercept is an estimate of the date of the root of the tree and *R*^2^ indicates the degree to which sequence evolution has been clocklike. However, the root-to-tip regression method is not statistically suitable for proper hypothesis testing because the individual data points are not independently distributed, and are instead partially correlated due to their phylogenetic shared ancestry (Drummond *et al.*, 2003a). To overcome this limitation, Navascués *et al.* (2010) suggested a non-parametric approach using permutations to test whether the correlation is stronger than expected if the sampling dates were randomly assigned. More recently, other phylogenetic approaches such as the date-randomization test (Duchêne *et al.*, 2015; Duffy and Holmes, 2009; Murray *et al.*, 2016; Ramsden *et al.*, 2008) or model selection/comparison (Duchene *et al.*, 2019; Murray *et al.*, 2016; Rambaut, 2000), although way more computationally intensive, have also been introduced and shown to be more robust tests for temporal signal detection and characterization.

**Fig. 1.**
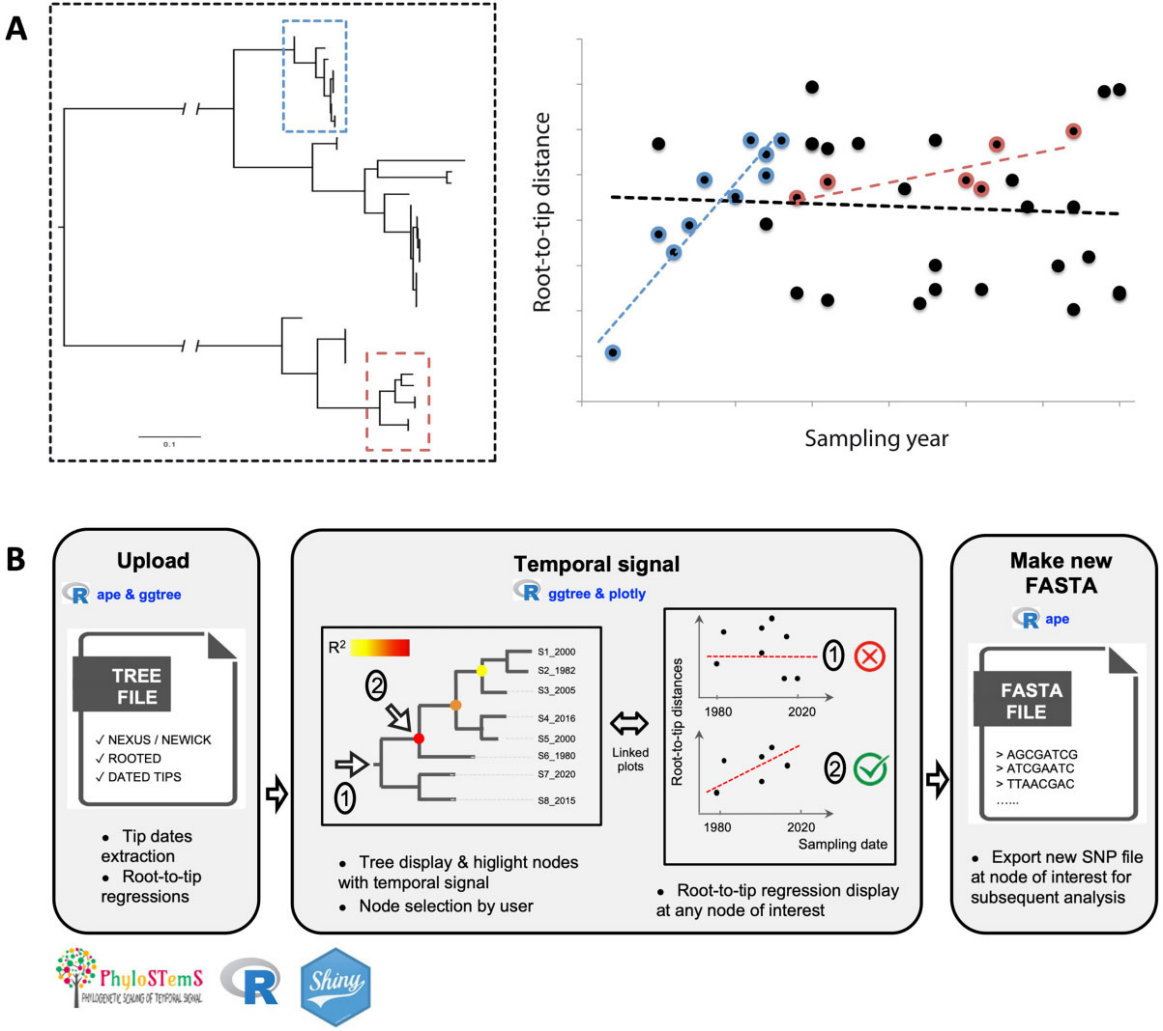
Phylostems rationale and workflow. (**A**) Let’s a tree (left panel) be constructed from a dataset of heterochronous sequences. When investigating temporal signal on the whole tree using the regular root-to-tip regression method (right panel), no significant signal is found as illustrated by the non-positive regression slope (black dotted line). Hence, tip-based inferences should not be performed at the whole tree timescale. However, as illustrated by the red and blue positive regression slopes calculated on two clade subsets (red and blue squares on the tree), positive temporal signal might exist at reduced timescales at which thorough tip-based inferences may be performed. The main objective of Phylostems is to provide the user with a graphical tool to detect without a priori such clades of interest. (**B**) Schematic representation of Phylostems workflow. Main boxes (‘Upload’, ‘Temporal signal’ and ‘Make new FASTA’) represent the internal structure of the application organized in three main panels. Major tasks performed in each panel are summarized along with sourced R packages

Despite its statistical pitfalls, the regression method remains a very helpful exploration tool to quickly assess the extent of temporal signal within a dataset. It only requires a rooted molecular phylogeny (whose branch lengths represent genetic distance) estimated from heterochronous (dated) sequences and runs instantaneously. Previously implemented in the popular and interactive graphical program TempEst (Rambaut *et al.*, 2016), the main limitation of the regression method relies in the fact that the temporal signal can only be tested at the whole-dataset (tree) scale. However, although a significant positive correlation would indicate the presence of detectable amounts of *de novo* mutations within a tree timescale, a non-positive (or a statistically non-significant) correlation does not necessarily mean that no temporal signal exists at a reduced timescale, as illustrated in Figure 1A. To fill this methodological gap, we introduce Phylostems, a new graphical and user-friendly tool developed to investigate temporal signal at every clade of a phylogenetic tree. Phylostems allows detecting without a priori whether any subset of a tree would contain sufficient temporal signal for tip-based inference to be performed. We provide a ‘how to’ guide by running Phylostems on an empirical dataset and supply insights on interpreting the outputs.

## 2 Phylostems software

### 2.1 General description

Phylostems (Phylogenetic Scaling of Temporal Signal) is an open source, graphical Shiny-based R application (Chang *et al.*, 2018; R Core Development Team, 2020) built for exploring temporal signal at various scales within a phylogenetic tree. Shiny is an R package that makes it easy to build interactive web applications from R (https://shiny.rstudio.com/). Phylostems can be either used online at https://pvbmt-apps.cirad.fr/apps/phylostems/ or executed locally by downloading its source code from https://gitlab.com/cirad-apps/phylostems. A schematic representation of Phylostems workflow is presented in Figure 1B. As input, Phylostems requires a rooted phylogenetic tree in computer-readable Nexus or Newick format with branch lengths scaled as genetic distances only, such as the ones computed using maximum-likelihood algorithms. In its current implementation, the online version of Phylostems allows uploading trees with 1500 sequences at maximum. Larger trees will need to be processed locally by sourcing the gitlab version. Importantly, sampling/isolation dates need to be known for each sequence and specified within tip labels. Before-Christ (B.C.) dates sometimes required to handle sequences generated from ancient DNA data can be specified using negative values (e.g. −400.5). Note that since missing dates are not allowed, sequences with unknown sampling years need to be pruned out from the tree prior to be uploaded in Phylostems.

**Fig. 2.**
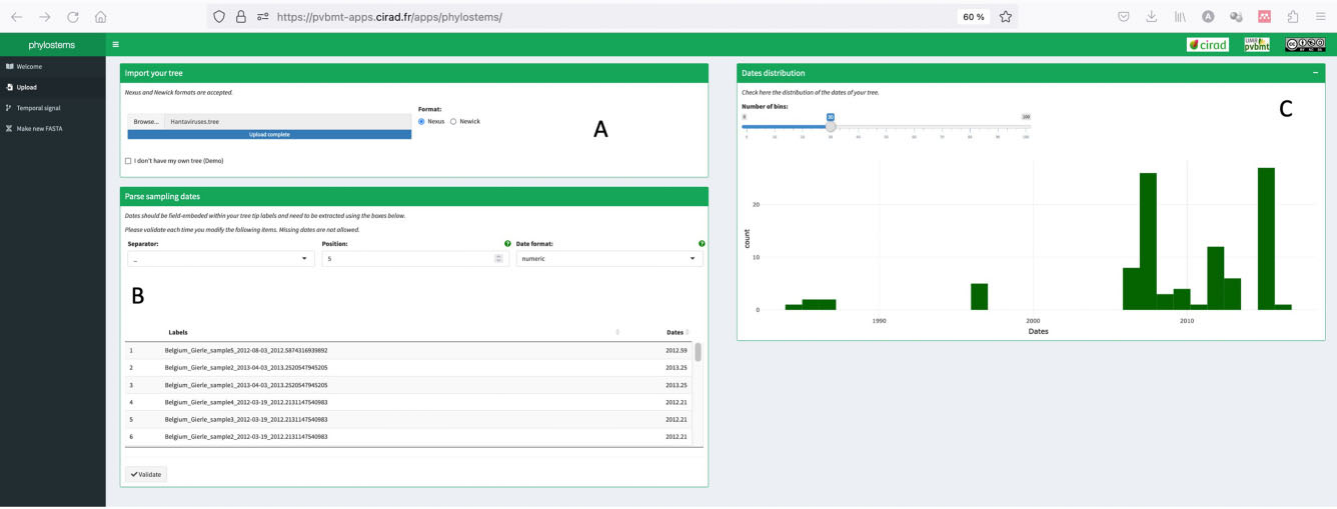
Phylostems’s upload panel requesting the user to load a phylogenetic tree (**A**) and specify tip sampling dates from field-embedded values (**B**). Once loaded, a distribution of sampling dates is plotted allowing for a visual check of sequences temporal width (**C**)

When a tree has correctly been loaded, a distribution of sampling dates is plotted within the ‘upload’ panel allowing for a visual check of sequences temporal width (Fig. 2C). Temporal signal is hence tested at every node of the input tree (including its root) meeting the following conditions required to perform a linear regression: *(i)* the node must be the parent of at least *n* = 3 tips, *(ii)* there should be at least *n* = 3 distinct combination of root-to-tip distances and sampling dates and *(iii)* there should be at least *n* = 2 different sampling dates. At each nodes meeting the above conditions, linear regression between sampling dates and root-to-tip distances is performed and the following parameters: (1) *p*-value, (2) slope, (3) adjusted *R*^2^ and (4) intercept with the *x*-axis values are recorded.

Phylostems’s main results are provided within the ‘Temporal signal’ panel. First, an annotated phylogenetic tree is interactively plotted by sourcing both ggtree and plotly R packages (Sievert, 2020; Yu *et al.*, 2017). On this tree, nodes with temporal signal, that is, nodes at which root-to-tip linear regression yielded a statistically significant and positive slope, are highlighted with colours scaling to *R*^2^ value. The default threshold for the linear regression *p*-value has been fixed to 0.05 but the user can interactively modify it using a slider bar, which enables easy investigation of nodes with borderline significant trends. A table summarizing the nodes with temporal signal is also displayed along with respective number of descending sequences, *p*-value, slope and adjusted *R*^2^ values. Most importantly, Phylostems allows the user to visualize the root-to-tip regressions at any chosen node of interest. To do so, one simply needs to click on a node and the associated root-to-tip regression will be displayed. Both the tree and the root-to-tip regression plots are linked, so that data points (or tree tips) selected in one plot will automatically be highlighted on the other one. This enables easy investigation of outliers and sequences or clades of interest.

Finally, when temporal signal is found at a specific node in the tree, Phylostems’s ‘Make new FASTA’ panel allows generating a new subset sequence FASTA file that only include the variant sites for the descending tips of this node, a dataset suitable for further tip-dating inferences.

### 2.2 How to guide using an empirical dataset

**Fig. 3.**
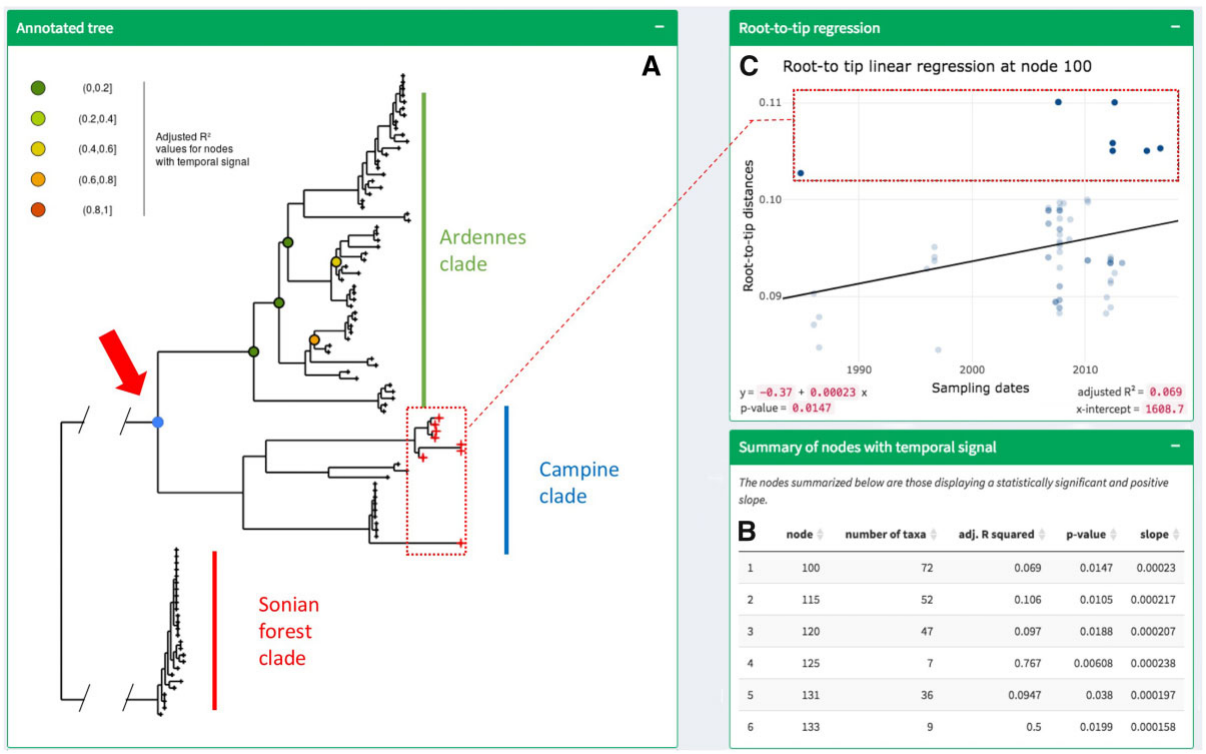
Phylostems results for the Hantaviruses dataset. (**A**) Annotated phylogenetic tree. Coloured circles indicate nodes at which temporal signal was found. (**B**) Summary table listing nodes with temporal signal and their associated statistical parameters. (**C**) Root-to-tip regression at node highlighted by the red arrow. Both the tree and the regression plots are linked, so that data points (or tree tips) selected in one plot will automatically be highlighted on the other one, as illustrated by the red-dotted frames

In the following, we use a previously published empirical dataset of 98 hantaviruses isolates sampled from bank voles in Belgium between 1984 and 2016 (Laenen *et al.*, 2019) to illustrate how Phylostems allows exploring temporal signal within phylogenetic trees. We downloaded from the original publication a rooted-ML tree file built from non-recombining genomic sequences and loaded it in Phylostems. Visual inspection of the Hantaviruses tree demonstrated heterogeneous temporal signal among clades, here referring to three geographical sampling areas namely Ardennes, Campine and Sonian Forest (Fig. 3A). Phylostems revealed a lack of temporal signal both at the whole tree scale and for the Sonian Forest clade. Temporal signal was observed at the MRCA of the Campine and Ardennes clades as well as within the Ardennes clade, as represented by the highlighted nodes on the tree. A table listing all the nodes associated with temporal signal along with their associated statistics is given in Figure 3B. When plotting the root-to-tip regression at the MRCA of the Campine and Ardennes clades, Phylostems allows visually identifying outlier samples that are significantly deviating from the root-to-tip regression line (Fig. 3C). Here, those felt within the Campine clade, suggesting that phylogenetic tip-based inferences should probably not be performed on both the Campine and Ardennes clades simultaneously. Possible causes for such outliers are multiple and will be discussed in the following section.

## 3 Discussion and conclusion

We introduce Phylostems, a new graphical and user-friendly tool developed to investigate temporal signal within phylogenetic trees using the root-to-tip regression method. Previous implementations of this method, such as in the popular and interactive graphical program TempEst (Rambaut *et al.*, 2016), were designed to test temporal signal at the whole tree scale (i.e. at its root). Investigating temporal signal at smaller phylogenetic scales was previously doable, but this task required the user to *(i)* a priori decide at which clade (i.e. samples) performing the test and *(ii)* manually splitting or reconstructing the tree for every of such clades. The main improvement of Phylostems is to allow detecting, in a single step and without a priori, any clade at which temporal signal may exist within a phylogenetic tree.

As illustrated by the empirical hantaviruses dataset analysed, temporal signal may sometimes be heterogeneous within a tree with substantial differences between clades. In such cases, we hope that Phylostems will help researchers detecting the most appropriate scales, if any, at which thorough tip-based inferences may be performed. However, because of the statistical pitfalls associated with the root-to-tip regression method (Rambaut, 2000; Rambaut *et al.*, 2016), Phylostems should rather be seen as a fast, visual and qualitative data exploration tool for temporal signal detection but should not be used to test hypotheses or undertake statistical model selection. Once temporal signal has been detected in Phylostems, we advise users to make use of other available methods such as non-parametric permutations (Navascués *et al.*, 2010), date-randomization test (Duchêne *et al.*, 2015; Duffy and Holmes, 2009; Murray *et al.*, 2016; Ramsden *et al.*, 2008) or model selection/comparison (Duchene *et al.*, 2019; Murray *et al.*, 2016) to validate the existence of MEPs in their datasets.

Phylostems can also help identifying outliers or groups of samples that substantially differ from the root-to-tip regression line and may require careful handling to avoid bias during phylogenetic inferences. First, as illustrated by the analyse of the Hantaviruses dataset, different clades or populations in a tree may be characterized by positive but contrasted root-to-tip regression patterns that might arise from sampling bias or differences in life-history traits between clades (e.g. environmental factors, population density, evolutionary rates or epidemiological parameters). In such a case, it is suggested to perform independent phylogenetic inferences on each clade/population (Laenen *et al.*, 2019). In other cases, outlier sequences whose sampling date is incongruent with their genetic divergence and phylogenetic position can be spotted from the regression plot (Rambaut *et al.*, 2016). Such anomalies can reflect a problem with *(i)* the sequence itself (e.g. low quality, sequencing/assembly/alignment errors, recombination or hypermutation) or *(ii)* the sampling date(s) (e.g. mislabelling or biological contamination). Should the case of such outlier sequences arise, those samples should be excluded from subsequent phylogenetic inferences.

Considering the impressive increase in availability and use of heterochronous datasets, we hope the functionality provided by Phylostems will help users to perform thorough tip-dating inferences. Phylostems is a dynamic application by nature. New functions will be added as new needs arise.

## Data Availability

Phylostems can be executed online at https://pvbmt-apps.cirad.fr/apps/phylostems/ but source code can also be downloaded from https://gitlab.com/cirad-apps/phylostems for local implementation. The Hantaviruses empirical tree used in this article is accessible from the gitlab repository.
